# Implication of different replicons in the spread of the VIM-1-encoding integron, In110, in Enterobacterales from Czech hospitals

**DOI:** 10.3389/fmicb.2022.993240

**Published:** 2023-01-04

**Authors:** Ibrahim Bitar, Costas C. Papagiannitsis, Lucie Kraftova, Vittoria Mattioni Marchetti, Efthymia Petinaki, Marc Finianos, Katerina Chudejova, Helena Zemlickova, Jaroslav Hrabak

**Affiliations:** ^1^Department of Microbiology, Faculty of Medicine, University Hospital in Pilsen, Charles University, Pilsen, Czechia; ^2^Biomedical Center, Faculty of Medicine, Charles University, Pilsen, Czechia; ^3^Department of Microbiology, University Hospital of Larissa, Larissa, Greece; ^4^National Reference Laboratory for Antibiotics, National Institute of Public Health, Prague, Czechia; ^5^Department of Medical Microbiology, 3rd Faculty of Medicine, Charles University, Prague, Czechia

**Keywords:** Enterobacterales, whole-genome sequencing, *bla*_VIM-1_, *bla*_VIM-4_, In110, plasmids, integrons

## Abstract

**Background:**

VIM metallo-β-lactamases are enzymes characterized by the ability to hydrolyze all β-lactams. Usually, *bla*_VIM_-like genes are carried by class 1 integrons. In the Czech Republic, only sporadic cases of VIM-producing Enterobacterales have been reported in which those isolates carried the VIM-1 carbapenemase-encoding integron In110. However, during 2019–2020, an increased number was reported. Therefore, the aim of the current study was to characterize the genetic elements involved in the increased spread of *bla*_VIM_ genes.

**Materials and methods:**

32 VIM-producing Enterobacterales collected between 2019 and 2020 were subjected to: antimicrobial susceptibility testing, integron analysis, and short reads sequencing. Based on the results, 19 isolates were selected as representative and sequenced using Sequel I platform.

**Results:**

The 32 VIM-producing isolates exhibited variations in the MICs of carbapenems. Based on short-read data, 26 of the 32 sequenced isolates harbored the *bla*_VIM-1_ allele while six isolates carried the *bla*_VIM-4_ gene. The most prevalent was the In110 integron (*n* = 24) and two isolates carried the In4873 class 1 integron. The *bla*_VIM-4_ allele was identified in class 1 integrons In1174 (*n* = 3), In416 (*n* = 1), In2143 (*n* = 1) and In2150. Long reads sequencing revealed that the *bla*_VIM_ was carried by: pKPC-CAV1193-like (*n* = 6), HI1 (pNDM-CIT; *n* = 4), HI2 (*n* = 3), FIB (pECLA; *n* = 2) and N (*n* = 1) incompatibility groups. Two *bla*_VIM_-carrying plasmids could not be typed by the database, while another one was integrated into the chromosome.

**Conclusion:**

We observed the spread of VIM-encoding integrons, mainly of In110, among Enterobacterales isolated from Czech hospitals, but also an increased number of novel elements underlining the ongoing evolution.

## Introduction

Mobile elements, such as integrons, transposons and plasmids, have played an important role in the spread of antimicrobial resistance genes among Enterobacterales. Integrons are genetic elements able to acquire and express genes in the form of cassettes. They can move due to their association with insertion sequences, transposons and plasmids (Mazel and Davies, 1999). Class 1 integrons, which are the most common integrons among clinical isolates, have been involved in the dissemination of more than 60 different gene cassettes conferring resistance to almost all antimicrobial categories (Mazel, 2006). One of the most recently characterized cassettes encodes VIM metallo-β-lactamases (MβLs), which are enzymes characterized by the ability to hydrolyze all β-lactams, including carbapenems (Laraki et al., 1999). Usually, *bla*_VIM_-like genes are carried by class 1 integrons, like In-e541 identified in Greece (Miriagou et al., 2003), In110 and In113 in Spain (Tato et al., 2010) or In416 in Italy (Colinon et al., 2007).

In the Czech Republic, only sporadic cases of VIM-producing Enterobacterales have been reported (Papousek et al., 2017; Papagiannitsis et al., 2018), from 2011 till 2015. Interestingly, those isolates carried the VIM-1 carbapenemase-encoding integron In110 (*bla*_VIM-1_-*aacA4*-*aadA1*; Lombardi et al., 2002). However, during 2019–2020, an increased number of VIM-producing Enterobacterales was isolated from Czech hospitals. Therefore, the aim of the current study was to characterize the genetic elements involved in the increased spread of *bla*_VIM_ genes, and to examine if In110 was the only/main integron associated with the expression of VIM carbapenemases.

## Materials and methods

### Bacterial isolates, susceptibility testing and confirmation of carbapenemase production

In 2019 and 2020, Czech hospitals referred a total of 32 VIM-producing Enterobacterales isolates with a meropenem MIC of >0.125 μg/ml (2012; using *E.coli* ATCC 25922 as a quality control strain) to the National Reference Laboratory for antibiotics. However, five of those isolates have been previously published as they carried an *mcr*-like gene (Bitar et al., 2020). Species identification was confirmed by matrix-assisted laser desorption ionization-time of flight mass spectrometry (MALDI-TOF MS) using MALDI Biotyper software (Bruker Daltonics, Bremen, Germany). All isolates were tested for carbapenemase production by the MALDI-TOF MS meropenem hydrolysis assay (Rotova et al., 2017). Additionally, the presence of carbapenemase-encoding genes (*bla*_KPC_, *bla*_VIM_, *bla*_IMP_, *bla*_NDM_, and *bla*_OXA-48_-like) was confirmed by PCR amplification (Poirel et al., 2004; Ellington et al., 2007; Naas et al., 2008; Yong et al., 2009). PCR products were sequenced as described below. Isolates positive for *bla*_VIM_-like genes were further studied.

Antimicrobial susceptibility was performed using broth microdilution according to European Committee on Antimicrobial Susceptibility Testing (EUCAST) (2012) guidelines. Susceptibility data were interpreted according to the criteria (version v12.0) of the EUCAST.^1^

### Integron analysis

Variable regions of class 1 integrons with *bla*_VIM_-like genes were amplified in two parts, from the 5΄conserved segment (5΄CS) to carbapenemase-encoding cassette and from carbapenemase-encoding cassette to the 3΄CS (Papagiannitsis et al., 2013). Whole-gene arrays were sequenced using an ABI 3500 sequencer (Applied Biosystems, Foster City, CA). The integron database, Integrall^2^ (Moura et al., 2009) was used to analyze and assign integron sequences.

### Short-read whole genome sequencing

All VIM-producing Enterobacterales were sequenced, using the Illumina MiSeq platform (Illumina Inc., San Diego, CA, United States). The genomic DNAs of the clinical isolates were extracted using the DNA-Sorb-B kit (Sacace Biotechnologies S.r.l., Como, Italy). Multiplexed DNA libraries were prepared using the Nextera XT library preparation kit, and 300-bp paired-end sequencing was performed on the Illumina MiSeq platform (Illumina Inc., San Diego, CA, United States) using the MiSeq v3 600-cycle reagent kit. Initial paired-end reads were quality trimmed using the Trimmomatic tool v0.33 (Bolger et al., 2014) and then, assembled by use of the de Bruijn graph-based *de novo* assembler SPAdes v3.14.0 (Bankevich et al., 2012).

### Long-read whole genome sequencing

Based on the results of short-read sequencing (see below), 19 VIM producers were selected to for long-read sequencing, to help close the whole plasmid sequences. These isolates were selected as representatives of all different hospitals, bacterial species, STs, replicon profiles and *bla*_VIM_ alleles.

Genomic DNA was extracted from the clinical isolates using NucleoSpin Microbial DNA kit (Macherey–Nagel, Germany). Whole genome sequencing (WGS) was performed on the Sequel I platform (Pacific biosciences, Menlo Park, CA, United States). Microbial multiplexing protocol was used for the library preparation according to the manufacturer instructions for Sheared DNA. DNA shearing was performed using the Megaruptor 2 (Diagenode, Liege, Belgium) using long hydropores producing 10 kb long inserts. No size selection was performed during the library preparation. The Microbial Assembly pipeline offered by the SMRT Link v9.0 software was used to perform the assembly and circularization with minimum seed coverage of 30X. Assembled sequences were annotated using the NCBI Prokaryotic Genome Annotation Pipeline (PGAP).

### Nucleotide sequence accession numbers

The nucleotide sequences of the genomes and plasmids were deposited and are available in GenBank (Supplementary Table S1) under the BioProject number PRJNA772913.

## Results

### VIM-producing Enterobacterales

During 2019–2020, a total of 32, Enterobacterales isolates with a meropenem MIC of >0.125 μg/ml were referred to the National reference laboratory for antibiotics from 15 laboratories. Among them, 23 isolates were identified to be *Enterobacter cloacae* complex, 5 were identified to be *Citrobacter freundii*, and 3 were identified to be *Klebsiella pneumoniae*. The one remaining VIM-producing isolate belonged to the bacterial species *Klebsiella michiganensis*.

All 32 VIM-producing isolates exhibited resistance to piperacillin, piperacillin-tazobactam and cephalosporins, while the variations in the MICs of aztreonam and carbapenems that were observed (Supplementary Table S1) might reflect the presence of additional resistance mechanisms in some of the isolates. Twenty-six of the VIM-producing isolates also exhibited resistance to tetracycline, 25 were resistant to chloramphenicol, 23 were resistant to gentamicin, 19 were resistant to ciprofloxacin, 3 were resistant to tigecycline, 6 were resistant to amikacin, whereas three of the isolates were resistant to colistin.

### Analysis of short-read sequencing results and VIM-encoding integrons

**Figure 1:**
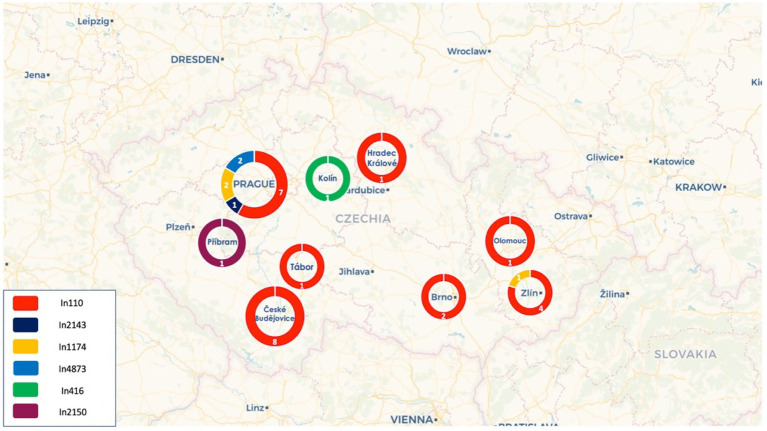
Distribution of the different integrons across the cities in the Czech Republic.

**Figure 2:**
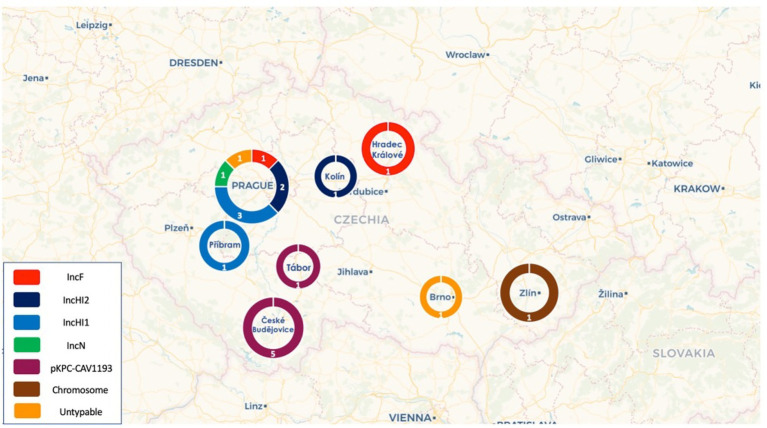
Distribution of the different plasmid types across the cities in the Czech Republic.

Based on short-read data, 26 of the 32 sequenced isolates harbored the *bla*_VIM-1_ allele (Tables 1, 2), while the remaining six isolates carried the *bla*_VIM-4_ gene. The *bla*_VIM-4_ gene was identified among 3\u00B0*C. freundii*, 1 *K. pneumoniae* and 2 *E. hormaechei* isolates. Moreover, characterization of the regions flanking the VIM-encoding genes by PCR mapping and sequencing data showed that *bla*_VIM_-like genes were located in six main types of class 1 integrons (Figures 1, 2). The most prevalent was the In110 integron identified in 24 VIM-1-producing isolates. The two remaining VIM-1-producing isolates, which belonged to *K. pneumoniae* ST54, carried the In4873 class 1 integron. In4873 integron, which is an In416-like element identified for the first time in Greece (Papagiannitsis et al., 2016), included the *bla*_VIM-1_, *aacA7*, *dfrA1*, *aadA1* and *smr2* gene cassettes. On the other hand, the *bla*_VIM-4_ allele was identified in class 1 integrons In1174 (*n* = 3), In416 (*n* = 1), In2143 (*n* = 1) and In2150 (Tables 1, 2). The class 1 integron In1174 includes an array of *aacA4* and *bla*_VIM-4_ gene cassettes. The In416 element, which was firstly reported in Italy (Colinon et al., 2007), comprises *bla*_VIM-4_, *aacA7*, *dfrA1*, *aadA1* and *smr2* gene cassettes. Additionally, the In2143, which was a novel class 1 integron carrying *bla*_VIM-4_, *aacA7* and *aacC2c* gene cassettes, was found in a ST108 *E. hormaechei* isolate. Finally, the novel integron In2150, which comprised *bla*_VIM-4_, *aacA7*, *smr2* cassettes, was identified in ST674 *C. freundii* isolate (Cfr-56322cz). Beside species-specific chromosomal β-lactamases, most of the clinical isolates also carried genes encoding TEM-1 penicillinases (*n* = 14) and/or OXA-1 oxacillinases (*n* = 13). Nine out of 22 *Enterobacter* isolates harboured the *bla*_CTX-M-15_ gene, while the *bla*_SHV-12_ gene was found among 2 isolates. Additionally, 2 out of 3 *K. pneumoniae* co-carried the carbapenemase-encoding gene *bla*_KPC-2_. All sequenced isolates exhibited a wide variety of resistance genes conferring resistance to aminoglycosides, sulfonamides, trimethoprim, streptomycin, fosfomycin (low-level resistance), fluoroquinolones, chloramphenicol, tetracyclines, colistin, erythromycin and/or rifampicin (Tables 1, 2).

**Table 1:** WGS data of the 19 isolates sequenced using both short (illumina) and long reads sequencing platform (PacBio).

**Isolate**	**Species**	**ST**	**VIM plasmid size**	**Inc**	**Integron type**	**Other replicons**	**Resistance genes**
48212*	*E. cloacae complex*	106	55,220	pKPC-CAV1193-like	In110	Col(pHAD28), IncFIB(pECLA), IncHI2	*mcr*-*9*, *aac*(*6′*)-*IIc*, *aadA2b*, *aph*(6)-*Id*, *dfrA19*, *catA2*, *sul1*, *sul2*, *tetD*, *aac*(*6′*)-*Ib*-*cr*, *qnrA1*, *ere*(*A*), *bla*_SHV_-12, *bla*_TEM-1b_
							*qnrS1*, *bla*_TEM-1a_, *bla*_VIM-1_, *aac*(*6′*)-*Ib3*
**48411**	*E. hormaechei*	1734	171765	IncFIB(pECLA)	In110	Col(pHAD28)	*aacA4, aadA1, dfrA14, bla*_VIM-1_ *, catA2, bla*_VIM-1B_ *, strA, strB, tetA, qnrS1,qacE, sul1, sul2, fosA*
48880*	*E. cloacae complex*	764	2,62,616	IncHI2	In416	Col(pHAD28), IncFIB(pECLA), IncFII(pECLA), IncR	*mcr*-*9.2*, *aac*(*6′*)-*II*, *aadA22*, *dfrA1*, *sul1*, *tetA*, *bla*_VIM-4_
48946*	*E. cloacae complex*	106	55,222	pKPC-CAV1193-like	In110	Col(pHAD28), IncFIB(pECLA), IncHI2	*mcr*-*9*, *aac*(*6′*)-*IIc*, *aadA2b*, *aph*(*3″*)-*Ib*, *aph*(6)-*Id*, *dfrA19*, *catA2*, *sul1*, *sul2*, *tetD*, *ere*(*A*), *bla*_TEM-1b_, *bla*_SHV-12_
							*aac*(*6′*)-*Ib3*, *qnrS1*, *bla*_TEM-1a_, bla_VIM-1_,
**48947**	*E. hormaechei*	190	55220	pKPC_49790_VIM_1	In110	IncHI2	*aacA4 (n=2), aac(3)-IIa, aadA1, aadA2b, catA1, catB3, dfrA14, bla*_VIM-1_ *, bla*_CTX-M-15_ *, bla*_OXA-1_ *, bla*_TEM-1A_ *, bla*_TEM-1B_ *, strA, strB, qnrB1, qnrS1, sul1, sul2, tetA*
**49589**	*E. hormaechei*	108	294454	IncHI2	In2143	IncFIB(pECLA), IncFII(pECLA), IncR	*aac(3)-Ib, aac(6')-Il, bla*_VIM-4_ *, qacE (n=2), qnrA1, sul1 (n=2), tetB, aacA7, aacC2c*
49790*	*E. cloacae complex*	106	55,220	pKPC-CAV1193-like	In110	Col(pHAD28), IncFIB(pECLA), IncHI2	*mcr*-*9*, *aac*(*6′*)-*IIc*, *aadA2b*, *aph*(*3″*)-*Ib*, *aph*(6)-*Id*, *dfrA19*, *catA2*, *sul1*, *sul2*, *tetD*, *aac*(*6′*)-*Ib*-*cr*, *qnrA1*, *ere*(*A*), *bla*SHV-12, *bla*_TEM-1b_, *aac*(*6′*)-*Ib3*, *qnrS1*, *bla*_TEM-1a_, *bla*_VIM-1_,
**54569**	*E. hormaechei*	92	171616	IncFIB (pECLA),IncFII (peCLA)	In110	IncQ1, Col440I, ColpVC	*aacA4, aac(3)-IIa, aac(6')-Ib3, aadA1, aph(3')-Via, bla*_VIM-1_*, bla*OXA-1*, catB3, dfrA14, fosA, qacE, sul1*
**57816**	*E. hormaechei*	106	55220	pKPC-CAV1193	In110	IncFIB (pECLA), IncHI2, Col (pHAD28)	*aacA4, aac(6')-Ib3, aac(6')-IIc, aadA2b (n=2),bla*_VIM-1_ *, bla*_TEM-1A_ *, bla*_TEM-1B_ *, bla*_SHV-12_ *, catA2, dfrA19, ere(A),mcr-9, qnrS1, fosA, qacE (n=3), strA, strB, sul1 (n=3), sul2, tet(D)*
**58983**	*E. cloacae*	421	64556	untypable	In110	IncFIB (pECLA)	*aacA4 (n=2), aac(3)-IIa, aadA1 (n=2), bla*_VIM-1_ *, bla*_CTX-M-15_ *, bla*_OXA-1_ *, bla*_TEM-1B_ *, catA1, catB3, dfrA14, qacE, qnrB1, strA, strB, sul1, sul2, tetA, fosA*
**59732**	*E. hormaechei*	1735	54956	pKPC-CAV1193	In110	IncFIB (pECLA), Col (pHAD28)	*aacA4, aadA2b, aac(6')-Ib3, bla*_VIM-1_*, bla*_TEM-1A_ *(n=2), qnrS1, fosA, qacE, sul1*
**60214**	*E. hormaechei*	92	311801	IncHI1 (pNDM-CIT)	In110	IncFIB (pECLA), IncFII (pECLA), Col (pHAD28)	*aacA4 (n=2), aac(6')-Ib3, aac(3)-IIa, aadA1 (n=2), aph(3')-Ia, catB3, bla_VIM-1_*, bla*_CTX-M-15_*, bla*_OXA-1_*, bla*_TEM-1B_* *, dfrA1, dfrA14, qnrB1, fosA, qacE, strA, strB, sul1, sul2, tet(A)*
51929*	*C. freundii*	95	3,69,945	IncHI2-/IncM1	In1174		*mcr*-*9*, *aac*(*6′*)-*II*, *aac*(3)-*I*, *aac*(*6′*)-*Ib3*, *ant*(*2″*)-*Ia*, *aadA1*, *aadA2b*, *aph*(*3′*)-*Ia*, *dfrA19*, *catA2*, *cmlA1*, *sul1*, *tetA*, *aac*(*6′*)-*Ib*-*cr*, *qnrA1*, *bla*_VIM-4_
**52323**	*C. freundii*	9	318136	IncHI1 (pNDM-CIT)	In110		*aacA4, aac(6')-Ib3, aadA1 (n=2), aph(3')-Ia, bla*_VIM-1_ *, catA1, dfrA1, qnrB75, qacE, sul1, tet(A)*
**56322**	*C. freundii*	674	344532	IncHI1A(NDM-CIT), IncHI1B(NDM-CIT)	In2150		*aacA7, aac(3)-IId, aadA2, bla*_VIM-4_, *bla*_TEM-1B_*, dfrA12, qnrB75, qacE, sul1 (n=2), sul2, tetA*
**56415**	*C. freundii*	673	106850	untypable	In1174	IncHI2	*aacA4, aac(6')-Ib3, aadA1, aadA2b (n=3), aadB (n=2), aph(3')-Ia (n=2), bla*_VIM-4_ *, catA2, cmlA1, dfrA19, mcr-9, qacE (n=4), sul-1 (n=4),*
**51135**	*K. pneumoniae*	11	chromosome	NA	In1174	IncFIB(K), IncM1	*aacA4, aac(6')-Ib3, bla*_VIM-1_ *,bla*_TEM-1B_ *, bla*_SHV-182_ *, qacE, cmx, oqxA, oqxB, fosA*
**59062**	*K. pneumoniae*	54	56199	IncN	In4873	IncFIB (K) (pCAV1099-114), IncH1B (pNDM-MAR),IncC, IncFIB (pQil) IncFII(K)	*aacA7, aac(6')-Im, aadA22, aph(3')-Ia, aph(2'')-Ib, bla*_VIM-1_ *, bla*_KPC-2_ *, catA1, dfrA1, oqxAB, qnrS1, fosA, qacE, sul1, tet(D)*
**53828**	*K. michiganensis*	226	364473	IncHI1 (pNDM-CIT)	In110	IncFII(Yp)	*aacA4, aph(3')-Ia, aadA1 (n=2), dfrA1, bla*_VIM-1_ *, catA1, qacE, sul1, tetA*

*These isolates were already published in Bitar et al. (2020).

**Table 2:** WGS data of the isolates sequenced only using short reads sequencing platform (illumina).

Isolate	Species	ST	Replicons	*bla*VIM-positive Integron	Resistance genes
49049	*E. cloacae*	92	IncFIB(pECLA), IncFII(pECLA), IncQ1, ColpVC, Col440I	In110	*aac(6′)-Ib-cr, aac(3)-IIa, aph(3′)-VIa, aadA1, bla*_VIM-1_ *, bla*_ACT-16_ *, catB3, dfrA14, sul1, fosA, qacE*
51524	*E. cloacae*	92	IncFIB(pECLA), IncFII(pECLA), IncQ1, ColpVC, Col440I	In110	*aac(6′)-Ib-cr, aadA1, aac(3)-IIa, aph(3′)-VIa, bla*_VIM-1_ *, bla*_OXA-1_ *, bla*_ACT-16_ *, catB3, dfrA14, sul1, qacE, fosA*
52089	*E. cloacae*	92	IncFIB(pECLA), IncFII(pECLA), IncHI1A(NDM-CIT), IncHI1B(NDM-CIT), Col(pHAD28)	In110	*aac(6′)-Ib-cr, aac(3)-IIa, aadA1, aph(3′)-Ia, bla*_VIM-1_ *, bla*_CTX-M-15_ *, bla*_OXA-1_ *, bla*_TEM-1B_ *, catB3, dfrA1, dfrA14, strA, strB, sul1, sul2, tetA, qnrB1, qacE*
54680	*E. asburiae*	25	IncFIB(pECLA), IncFII(pECLA), IncHI2, IncHI2A	In110	*aac(6′)-Ib-cr, aac(3)-IIa, aadA24, arr-3, bla*_VIM-1_ *, bla*_CTX-M-15_ *, bla*_OXA-1_ *, bla*_TEM-1B_ *, bla*_ACT-6_ *, catA1, catB3, dfrA14, strA, strB, sul1, sul2, tetA, qnrB1, qacE, fosA*
54818	*E. cloacae*	92	IncFIB(pECLA), IncFII(pECLA), IncHI2, IncHI2A, IncQ1, ColpVC	In110	*aac(6′)-Ib-cr, aac(3)-IIa, aph(3′)-VIa, aadA1, bla*_VIM-1_ *, bla*_CTX-M-15_ *, bla*_OXA-1_ *, bla*_TEM-1B_ *, bla*_ACT-16_ *, catA1, catB3, dfrA14, strA, strB, sul1, sul2, tetA, qnrB1, qacE*
54822	*E. cloacae*	92	IncFIB(pECLA), IncFII(pECLA), IncQ1, ColpVC, Col440I	In110	*aac(6′)-Ib-cr, aac(3)-IIa, aph(3′)-VIa, aadA1, dfrA14, bla*_VIM-1_ *, bla*_OXA-1_ *, bla*_ACT-16_ *, catB3, sul1, qacE, fosA*
55614	*E. cloacae*	92	IncFIB(pECLA), IncFII(pECLA), IncQ1, ColpVC, Col440I	In110	*aac(6′)-Ib-cr, aac(3)-IIa, aph(3′)-VIa, aadA1, bla*_VIM-1_ *, bla*_OXA-1_ *, bla*_ACT-16_ *, catB3, dfrA14, sul1, qacE, fosA*
56501	*E. cloacae*	190	IncHI2, IncHI2A, pKPC-CAV1193, Col(pHAD28)	In110	*aac(6′)-Ib-cr, aac(3)-IIa, aadA1, aadA2b, bla*_VIM-1_*, bla*_CTX-M-15_*, bla*_OXA-1_, *bla*_TEM-1B_*, bla*_ACT-7_*, catA1, catB3, dfrA14, strA, strB, sul1, sul2, tetA, qnrB1, qacE, fosA*
57689	*E. cloacae*	106	IncFIB(pECLA), IncFII(pECLA), IncHI2, IncHI2A, pKPC-CAV1193, Col(pHAD28)	In110	*aac(6′)-Ib-cr, aac(6′)-IIc, aph(6)-Id, aac(3)-IIa, aadA1, aadA2b, bla*_VIM-1_ *, bla*_CTX-M15_ *, bla*_OXA-1_ *, bla*_SHV-12_ *, bla*_TEM-1B_ *, bla*_ACT-15_ *, catA1, catB3, ereA, dfrA14, strA, strB, sul1, sul2, tetA, tetD, qnrB1, qnrS1, qacE, fosA*
61347	*E. cloacae*	1735	Col(pHAD28), pKPC-CAV1193, Col(pHAD28)	In110	*aac(6′)-Ib-cr, aadA2b, bla*_VIM-1_*, bla*_TEM-1A_*, bla*ACT-15*, sul1, qacE, fosA*
61503	*E. cloacae*	252	IncFIB(pECLA), IncFII(pECLA), repA(pENTd4a)	In110	*aac(6′)-Ib-cr, aac(3)-IIa, aadA1, bla*_VIM-1_ *, bla*_OXA-1_ *, bla*_ACT-3_ *, catBe, dfrA14, sul1, qnrE1, qacE, fosA1*
50714	*C. freundii*	673	IncFII(SARC14), IncN	In110	*aac(6′)-Ib-cr, aph(3″)-Ib, aadA1, bla*_VIM-1_ *, bla*_CMY-78_ *, dfrA14, sul1, sul2, qacE, qnrS1*
59343	*K. pneumoniae*	54	IncC, IncN, IncFIB(pQil), IncFII(K), IncFIB(K)(pCAV1099-114), IncHI1B(pNDM-MAR)	In4873	*aac(6′)-Im, aacA27, aph(3′)-Ia, aadA2, bla*_VIM-1_ *, bla*_KPC-2_ *, bla*_SHV-178_ *, catA1, dfrA1, sul1, tetD, qnrS1, qacE, oqxA, oqxB, fosA*

WGS data revealed that most of the isolates belonging to *E. cloacae* complex isolates belonged to sequence types ST92 (*n* = 8), ST106 (*n* = 5), and ST190 (*n* = 2; Tables 1, 2). The remaining seven *Enterobacter* isolates were ST25, ST92, ST108, ST252, ST421, ST764, and the novel STs 1734 and 1735. The isolates belonging to *C. freundii* species were assigned to ST95 (*n* = 2), ST9 (*n* = 1), and ST673 (*n* = 1) and ST674 (*n* = 1). ST673 and ST674 were novel STs. The *K. pneumoniae* isolates included two STs. The VIM-1 producers belonged to ST54, while the VIM-4-producing *K. pneumoniae* was ST11. Finally, the *K. michiganensis* (closely related to *K. oxytoca*) isolate was assigned to ST226.

### Localization of VIM-encoding integrons

Based on short-read data, 19 VIM-producing isolates were selected to be sequenced by the Sequel I platform, to close plasmid sequences. Analysis of long-read sequencing data revealed the presence of several *bla*_VIM_-carrying plasmid sequences belonging to different Inc. groups and presenting diverse sizes (Table 1). Based on PlasmidFinder analysis of plasmid sequences, 16 out of 19 *bla*_VIM_-carrying plasmids could be assigned tο: pKPC-CAV1193-like (*n* = 6), HI1 (pNDM-CIT; *n* = 4), HI2 (*n* = 3), FIB (pECLA; *n* = 2) and N (*n* = 1) incompatibility (Inc) groups (Figure 2). Two of the remaining *bla*_VIM_-carrying plasmids could not be typed by the database, while in the ST11 *K. pneumoniae* isolate the VIM-4-encoding integron, In1174, was integrated into the chromosome.

All pKPC-CAV1193-like plasmids (*n* = 6) carried the VIM-1 encoding integron In110. These plasmids, which were ~ 55,220-bp in size (except p59732CZ_VIM), were identical to plasmid p48212_VIM (Supplementary Figure S1) characterized previously from ST106 *E. hormaechei*, carrying *mcr-9* gene, isolated from a Czech hospital (Bitar et al., 2020). Plasmid p59732CZ_VIM lacked a 266-bp fragment in the ORF encoding a GNAT family N-acetyltransferase.

Also, three (p52323cz_VIM, p53828cz_VIM and p60214cz_VIM) out of four IncHI1 (pNDM-CIT) plasmids carried the In110. These plasmids were identical to VIM-1-encoding plasmid pLec-476cz (Supplementary Figure S2), which was previously characterized from a *Leclercia adecarboxylata* isolate (Papousek et al., 2017) recovered during a survey study focused on compliance with hand hygiene among the staff of a different Czech hospital in May 2011. On the other hand, the fourth IncHI1 (pNDM-CIT) plasmid (p56322_VIM), was carried by a ST674 *C. freundii* isolate. This plasmid harbored the VIM-4-encoding integron In2150. Plasmid p56322_VIM showed moderate similarity to IncHI1 plasmids, encoding VIM-1 (like p53828cz_VIM [72% coverage, 98.68% identity]), while the highest similarity was observed for the IncHI1 plasmid pRHBSTW-00135_2 (80% coverage, 100% identity; GenBank accession no. CP056828; Supplementary Figure S3) that was characterized from a wastewater influent sample collected in the United Kingdom. Of note was that plasmid pRHBSTW-00135_2 carried no resistance genes. p56322_VIM was composed of a partial IncHI1 backbone and a MDR region. Segments of the IncHI1 backbone, encoding proteins involved in the conjugative transfer system, were duplicated in the p56322_VIM plasmid. Furthermore, beside In2150, the MDR region contained the In27 integron, consisting of *dfrA12*, *gcuF* and *aadA2* gene cassettes, the *bla*_TEM-1_ and *aac*(3)*-IId* resistance genes, and regions conferring resistance to macrolides, mercury and chromate.

Two IncHI2 VIM-4-encoding plasmids (p48880_MCR_VIM and p51929_MCR_VIM), which also carried the *mcr-9* resistance gene, have been previously characterized (Bitar et al., 2020). The third IncHI2 plasmid, p49589_VIM, which was 294,454-bp in size, carried the novel VIM-4-encoding integron In2143. It exhibited moderate similarity to p48880_MCR_VIM (76% coverage, 99.48% identity) and p51929_MCR_VIM (76% coverage, 99.97% identity), while it was almost identical (99% coverage, 100% identity) to IncHI2 plasmid p48293_VIM (Supplementary Figure S4), which was previously sequenced from the *E. hormaechei* strain Ecl-48293co-producing KPC-2 and VIM-4 carbapenemases, during a study describing the ongoing spread of KPC-type producers in Czech hospitals (Kraftova et al., 2021). p49589_VIM was typed as sequence type 1 (ST1) following the IncHI2 pDLST scheme (Garcia-Fernandez and Carattoli, 2010). In agreement with other IncHI2 replicons, plasmid backbone was composed of regions responsible for replication (*repHI2*), conjugative transfer (*trh* genes), and plasmid maintenance (*par* gene). Additionally, similarly to other IncHI2 plasmids, it carried genes conferring resistance to tellurium (*terZABCDEF*), while genes conferring resistance to arsenic (*arsCBRH*) were not found. Moreover, one multidrug resistance (MDR) region was identified, in which the integron In2143 was embedded in a Tn*1696*-like transposon, also carrying a *qnrA1* resistance gene and a mercury (*mer*) resistance operon.

In IncFIB plasmids, p48411_VIM and p54569_VIM showed limited similarity (57% coverage, 100% identity) to each other (Supplementary Figure S5), despite being both typed as IncFIB (pECLA) by PlasmidFinder analysis. Plasmid p48411_VIM, which contained only the IncFIB replicon of pECL_A (Ren et al., 2010), seemed to be a fusion derivative of plasmids pLec-476cz and pECL_A. It contained a 46,500-bp segment (1–32,410 and 157,676–171,765) being identical to a sequence of pLec-476cz including a part of the plasmidic backbone and a part of the MDR region, which contained the class 1 integron In110. The remaining 125,265-bp sequence, which contained a region encoding a type-F conjugative transfer system, carried regions responsible for resistance to tellurium, copper and silver, and a second MDR region carrying *dfrA14*, *catA2*, *sul2*, *strA*, *strB* and *bla*_TEM-1_ resistance genes. This MDR region resembled the MDR region in pECL_A and p60214_IncFII. The pECL_A-like plasmid, p60214_IncFII, was sequenced from a ST92 *E. hormaechei* isolate, characterized during this study. On the other hand, plasmid p54569_VIM was a derivative of pECL_A, which acquired a Tn*1721*-like transposition module (9986–24,830 bp) carrying In110 (Supplementary Figure S5). An identical transposition module has also been observed in plasmids p58983_VIM and pEncl-30969cz (as seen below). Direct repeats of 5 bp (TCCGG) were found at the boundaries of the Tn*3*-like element, suggesting its transposition into the pECL_A-like backbone. Unlike p48411_VIM, no *tra* region was found on p54569_VIM. Additionally, it contained both IncFIB and IncFII replicons of pECL_A (Ren et al., 2010).

The IncN plasmid p59062_VIM, which carried the VIM-1-encoding integron In4873, was typed as ST7 based on plasmid MLST (pMLST) scheme for rapid categorization of IncN plasmids (Garcia-Fernandez et al., 2011). It showed extensive similarity with other IncN plasmids (Supplementary Figure S6), like pTE_C_1 (83% coverage, 99.99% identity; GenBank accession no.MW574936), pNL194 (81% coverage, 99.22% identity; Miriagou et al., 2010) and p3846_IncN_VIM-1 (88% coverage, 100% identity; Marchetti et al., 2021). The In4873 integron was inserted between the genes, encoding EcoRII endonuclease and resolvase, of the IncN plasmidic backbone. Furthermore, a Tn*21* fragment consisting of *tniB* and *tniA* was found next to the 3’CS, 108 bp downstream of *orf5*, as in In2-like integrons. The *qnrS1* resistance gene was also found in p50962_VIM, located downstream of *fipA*.

The non-typeable plasmid p58983_VIM, which was characterized from a ST421 *E. cloacae* complex isolate, carried the VIM-1-encoding integron In110. It comprised a plasmidic backbone which was identical (100% coverage, 100% identity) to plasmid p54569CZ_2 (characterized from a ST92 *E. hormaechei* isolate in this study; Supplementary Figure S7). Additionally, it contained a MDR region being identical to the respective regions of plasmids p54569_VIM (characterized from a ST92 *E. hormaechei* isolate in this study) and pEncl-30969cz (sequenced from a VIM-1-producing ST92 *E. cloacae* isolated, in 2015, in a Czech hospital; Papagiannitsis et al., 2018). Similar to pEncl-30969cz, the MDR of p58983_VIM was a Tn*1721*-like transposon structure containing In110, a Tn*21* fragment, a Tn*3*-like transposon, and a *qnrB*-like gene conferring resistance to quinolones (Halova et al., 2014). Two copies of an IS*5075* element, which was shown previously to target the IRs of Tn*21*-like transposons (Partridge and Hall, 2003), disrupted the IRs of the Tn*3*-like. The remaining part of the Tn*21 mer* module was probably deleted due to insertion of the Tn*3*-like transposon, but in contrast to pEncl-30969cz the Tn*3*-like was in an opposite orientation. Target site duplications of 6 bp (CAATAC) were found at the boundaries of Tn*1721*-like transposon, suggesting its transposition into the p58983_VIM backbone.

The non-typeable plasmid p56415_VIM, which carried the VIM-4-encoding integron In1174, was composed of two parts: the plasmidic backbone and the MDR region. The plasmidic backbone was identical to plasmid p51929, which was characterized from a VIM-4-encoding *C. freundii* isolate (Cfr-51929cz; Bitar et al., 2020). Even though the plasmid p51929 carried no resistance genes, Cfr-51929cz also harbored the VIM-4-encoding integron In1174 in the *mcr-9*-positive plasmid (p51929_MCR_VIM that belonged to IncHI2 group). The MDR region of p56415_VIM consisted of a Tn*3*-like element carrying the In1174 integron (Supplementary Figure S8). The same Tn*3*-like transposon was inserted into the chromosome of the *K. pneumoniae* isolate Kpn51135cz (as seen below). Unlike Kpn51135cz, the IRmer of the Tn*3*-like element was intact, whilst the IRtnp was disrupted by IS*5075*. Direct repeats of 6 bp (AATATG) were found at the boundaries of the integrated segment, suggesting its transposition into the p56415-VIM plasmid.

Finally, in *K. pneumoniae* isolate Kpn51135cz, the VIM-4-encoding integron In1174 was localized in a Tn*3*-like transposon element that was integrated into the *K. pneumoniae* chromosome (Supplementary Figure S9). The IR of the Tn*3*-like *tnp* module and the IR of the *mer* module, at the boundaries of the *K. pneumoniae* chromosome, were disrupted by two copies of the insertion sequences IS*4321*. Direct repeats of 5 bp (CTCAA) were found at the boundaries of the integrated segment, suggesting its transposition into the *K. pneumoniae* chromosome.

## Discussion

In the current study, we characterized 32 VIM-producing Enterobacterales (including *E. cloacae*, *C. freundii*, *K. pneumoniae* and *K. michiganensis* isolates), which were isolated during the period of 2019–2020, in order to analyze the genetic determinants involved in the dissemination of *bla*_VIM_ alleles in various Czech hospitals. Our findings showed the presence of two *bla*_VIM_ variants, *bla*_VIM-1_ (*n* = 26) and *bla*_VIM-4_ (*n* = 6), carried by a significant number of integrons. The main VIM-1-encoding integron, identified during this study, was In110 (*n* = 24), while the In4873 was found in two ST54 *K. pneumoniae* isolates. On the other hand, three VIM-4-producing isolates included the In1174 integron, one isolate carried the In416, and the two remaining isolates carried novel integron structures. The In110 integron has been previously reported from isolates of Czech origin (Papousek et al., 2017; Papagiannitsis et al., 2018). Additionally, the presence of other integron types and the emergence of novel integron structures demonstrates the ongoing evolution of genetic determinants involved in the spread of resistance. Integrons usually carry more than one resistance gene, conferring resistance to multiple antimicrobial classes. Therefore, integrons are associated with the emergence of MDR bacteria and the fact of co-selection.

**Figure 3:**
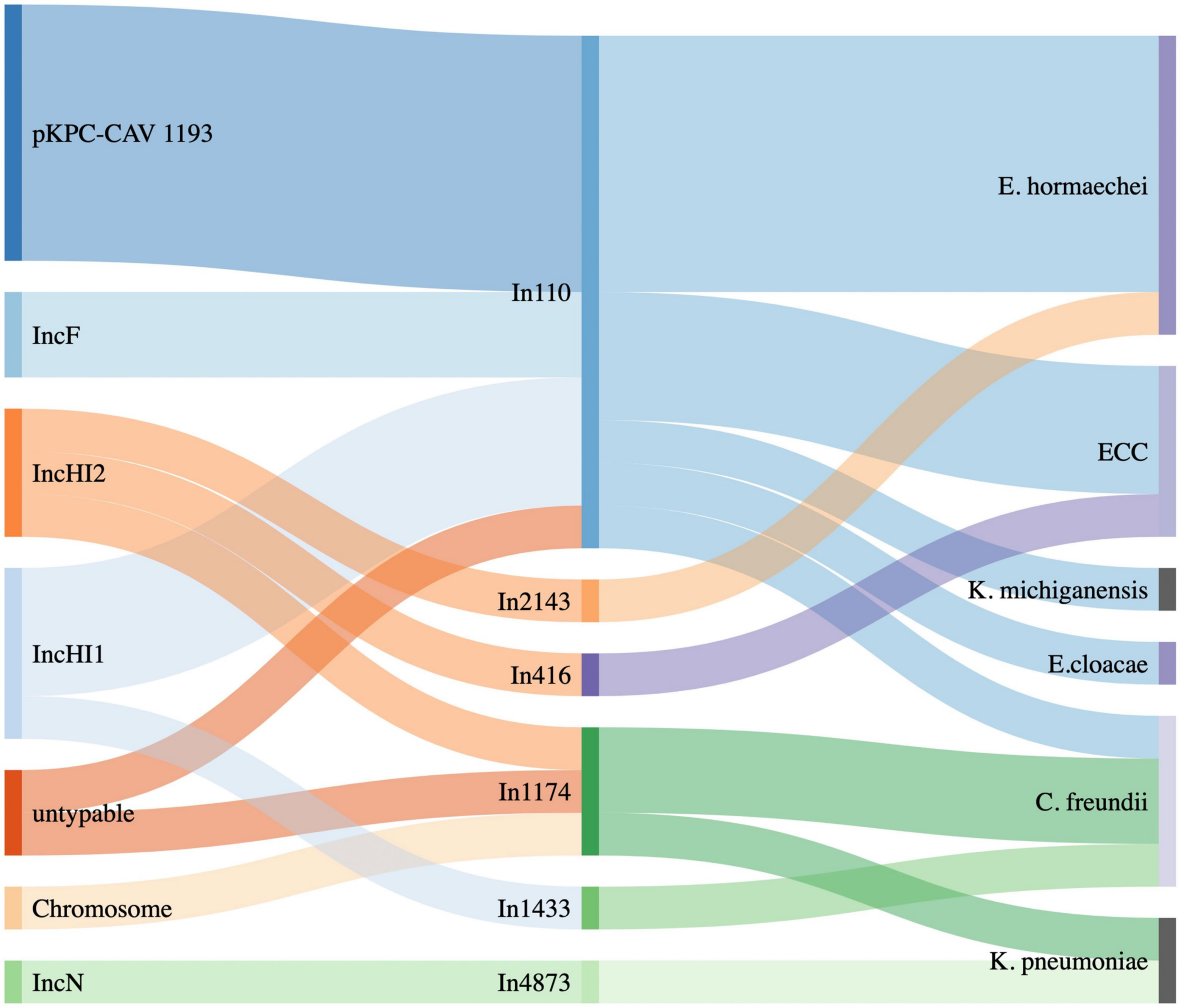
Sankey diagram showing the distribution of different integrons linking to the plasmid types (left) and to the bacterial species detected (right).

Moreover, the analysis of WGS data showed that *bla*_VIM_-positive integrons were carried by several plasmids belonging to different Inc. groups (pKPC-CAV1193-like, HI1, HI2, FIB, N and non-typeable) and presenting diverse sizes (Table 1; Figure 3). Also, in one *K. pneumoniae* isolate, belonging to ST11, the VIM-4-encoding integron, In1174, was integrated into the chromosome (Supplementary Figure S9). Another interesting finding is the emergence of hybrid plasmids, such as p48411_VIM and p54569_VIM. The plurality of different plasmids, carrying *bla*_VIM_ alleles, and the emergence of hybrid plasmids are two features widening the spectrum of species that these resistance determinants could be disseminated. Also, of note was the characterization of plasmids being identical to VIM-1-encoding plasmid pLec-476cz (Supplementary Figure S2), which was previously characterized from a *L. adecarboxylata* isolate (Papousek et al., 2017) recovered during a survey study focused on compliance with hand hygiene among the staff of a different Czech hospital in May 2011. This data is worrying, since it highlights the hidden source, and the continuous spread of resistance determinants, and especially of carbapenemase-encoding genes, in Czech hospitals.

**Figure 4:**
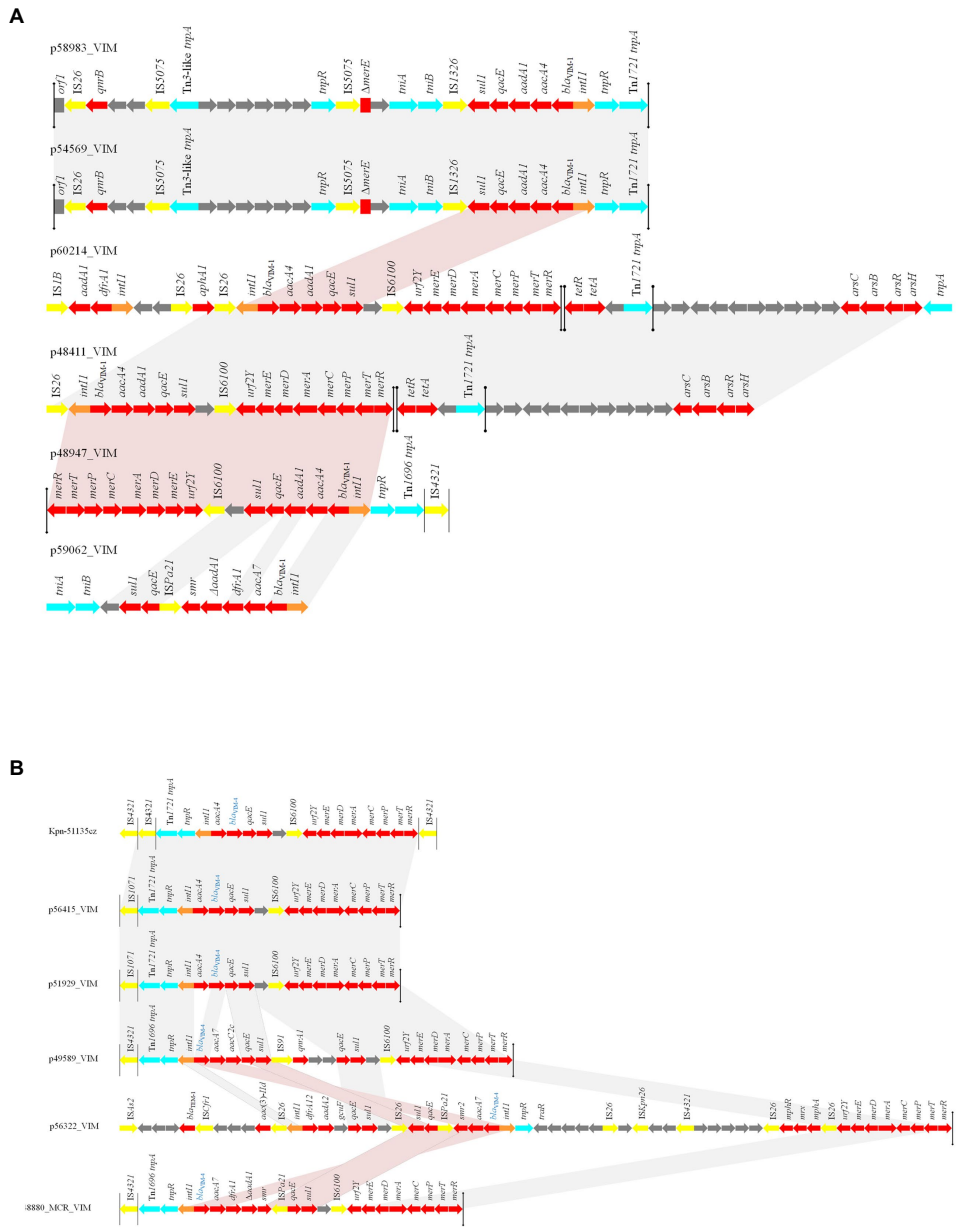
Linear comparisons of **(A)** VIM-1- **(B)** VIM-4- encoding MDR regions characterized from Enterobacterales isolated from Czech hospitals, during 2019–2020. Arrows show the direction of transcription of open reading frames (ORFs). Resistance genes are shown in red. IS elements and transposases are shown in yellow and aqua, respectively. *intI1* genes are shaded orange. The remaining genes are shown in gray. Homologous segments (representing ≥99% sequence identity) are indicated by light gray shading, while pink shading shows inverted homologous segments.

Finally, analysis of MDR regions revealed an increased divergence among these sequences (Figure 4). However, we observed the same VIM-1-encoding MDR region in IncHI1 (pNDM-CIT) plasmids (like p60214_VIM) and the IncFIB (pECLA) plasmid p48411_VIM, while a part of this structure was found in pKPC-CAV1193-like plasmids (like p48947_VIM). The presence of the same MDR region in plasmids of different Inc. groups may be the outcome of homologous recombination events. Also, we observed the presence of an identical transposition module in the IncFIB (pECLA) plasmid p54569_VIM and the non-typeable plasmid p58983_VIM. A totally different structure was identified in the IncN plasmid p59062_VIM. Regarding the VIM-4-encoding structures, we observed the presence of an identical transposition module in the IncHI2 plasmid p51929_MCR_VIM, the non-typeable plasmid p56415_VIM and in the chromosome of the ST11 *K. pneumoniae* isolate Kpn51135cz. A similar transposition module, differing by acquisition of a different VIM-4-encoding integron (In2143 in p49589_VIM unlike In1174 in p56415_VIM) and of *qnrA1* resistance gene, was found in IncHI2 plasmid p49589_VIM. The presence of the same transposition modules into different replicons indicates the functional role of the specific transposons. On the other hand, totally diverse MDR regions were identified in IncHI1 (pNDM-CIT) plasmid p56322 and IncHI2 plasmid p48880_MCR_VIM.

One limitation of the study is not investigating the ability of the detected plasmids to conjugate. This is due to the high diversity in plasmids detected and to the different conjugation dynamics between *in-vitro* and *in-vivo*. This difference is prevalent for example to IncR and pKPC-CAV1193-like plasmids. The *in-vitro* conjugation assay for these two plasmids detected has a very low conjugation rate if any (Sheppard et al., 2016; Bitar et al., 2020) yet epidemiological results state the high ability of these plasmids to conjugate.

Of note, all pKPC-CAV1193 plasmids carried the In110 integrons which seems to be spread in Cesky Budejovice implying an outbreak of this plasmid in the city (Figures 1, 2). Moreover, all IncF and most of IncHI1 plasmids (except for one plasmid carrying In1433 integron; Figure 3) also carried In110 integrons. The IncHI2 plasmids carried different types of integrons such as In2143, In416 and In1174 (which was also found on the chromosome and on one un-typable plasmid). Finally, IncN plasmid carried In4873. This shows, although there is dominance of In110, that there are multiple integrons responsible for the dissemination of *bla*_VIM_ irrespective of the plasmid type. Additionally, of interest is the association of In110 with multiple replicons (Figure 3).

## Conclusion

During this study, we observed the spread of VIM-encoding integrons, mainly of In110, among Enterobacterales isolated from Czech hospitals, in 2019 and 2020. Additionally, we noticed the presence of multiple mechanisms, including (i) the functional acquisition of *bla*_VIM_-carrying transposons, (ii) the acquisition of *bla*_VIM_-carrying MDR regions *via* homologous recombination events (iii) the ongoing evolution of *bla*_VIM_-carrying integrons, (iv) and the hidden spread of *bla*_VIM_-carrying replicons, involved in the emergence and spread of MDR regions carrying carbapenemase-encoding genes. Thus, ongoing surveillance of carbapenem-resistance is of utmost importance to control the spread of these emerging pathogens.

## Data availability statement

The datasets presented in this study can be found in online repositories. The names of the repository/repositories and accession number(s) can be found in the article/Supplementary material.

## Author contributions

IB and CP designed the study, analyzed the data, and wrote the manuscript. KC collected the samples. IB, LK, HZ, VM, EP, and MF conducted the experiments. IB and JH secured the funding. All authors contributed to the article and approved the submitted version.

## Funding

The study was supported by research project grant NU20J- 05-00033, provided by the Czech Health Research Council, and by the project National Institute of Virology and Bacteriology (Programme EXCELES, ID Project No. LX22NPO5103) - funded by the European Union - Next Generation EU.

## Conflict of interest

The authors declare that the research was conducted in the absence of any commercial or financial relationships that could be construed as a potential conflict of interest.

## Publisher’s note

All claims expressed in this article are solely those of the authors and do not necessarily represent those of their affiliated organizations, or those of the publisher, the editors and the reviewers. Any product that may be evaluated in this article, or claim that may be made by its manufacturer, is not guaranteed or endorsed by the publisher.

## Footnotes

^1^ http://www.eucast.org/

^2^ http://integrall.bio.ua.pt/

## References

[ref1] Bankevich A. Nurk S. Antipov D. Gurevich A. A. Dvorkin M. Kulikov A. S.. (2012). SPAdes: a new genome assembly algorithm and its applications to single-cell sequencing. J. Comput. Biol. 19, 455–477. doi: 10.1089/cmb.2012.0021, PMID: 22506599 PMC3342519

[ref2] Bitar I. Papagiannitsis C. C. Kraftova L. Chudejova K. Mattioni Marchetti V. Hrabak J. (2020). Detection of five mcr-9-carrying Enterobacterales isolates in four Czech hospitals. mSphere 5:e01008-20. doi: 10.1128/mSphere.01008-20 33298573 PMC7729258

[ref3] Bolger A. M. Lohse M. Usadel B. (2014). Trimmomatic: a flexible trimmer for Illumina sequence data. Bioinformatics 30, 2114–2120. doi: 10.1093/bioinformatics/btu170, PMID: 24695404 PMC4103590

[ref4] Colinon C. Miriagou V. Carattoli A. Luzzaro F. Rossolini G. M. (2007). Characterization of the IncA/C plasmid pCC416 encoding VIM-4 and CMY-4 beta-lactamases. J. Antimicrob. Chemother. 60, 258–262. doi: 10.1093/jac/dkm171, PMID: 17540674

[ref5] Ellington M. J. Kistler J. Livermore D. M. Woodford N. (2007). Multiplex PCR for rapid detection of genes encoding acquired metallo-beta-lactamases. J. Antimicrob. Chemother. 59, 321–322. doi: 10.1093/jac/dkl481, PMID: 17185300

[ref6] European Committee on Antimicrobial Susceptibility Testing (EUCAST). (2012). EUCAST guidelines for detection of resistance mechanism and specific resistances of clinical and/or epidemiological importance. European committee on antimicrobial susceptibility testing. Växjö, Sweden: EUCAST.

[ref7] Garcia-Fernandez A. Carattoli A. (2010). Plasmid double locus sequence typing for IncHI2 plasmids, a subtyping scheme for the characterization of IncHI2 plasmids carrying extended-spectrum beta-lactamase and quinolone resistance genes. J. Antimicrob. Chemother. 65, 1155–1161. doi: 10.1093/jac/dkq101, PMID: 20356905

[ref8] Garcia-Fernandez A. Villa L. Moodley A. Hasman H. Miriagou V. Guardabassi L.. (2011). Multilocus sequence typing of IncN plasmids. J. Antimicrob. Chemother. 66, 1987–1991. doi: 10.1093/jac/dkr225, PMID: 21653604

[ref9] Halova D. Papousek I. Jamborova I. Masarikova M. Cizek A. Janecko N.. (2014). Plasmid-mediated quinolone resistance genes in Enterobacteriaceae from American crows: high prevalence of bacteria with variable qnrB genes. Antimicrob. Agents Chemother. 58, 1257–1258. doi: 10.1128/AAC.01849-13, PMID: 24247140 PMC3910892

[ref10] Kraftova L. Finianos M. Studentova V. Chudejova K. Jakubu V. Zemlickova H.. (2021). Evidence of an epidemic spread of KPC-producing Enterobacterales in Czech hospitals. Sci. Rep. 11:15732. doi: 10.1038/s41598-021-95285-z, PMID: 34344951 PMC8333104

[ref11] Laraki N. Galleni M. Thamm I. Riccio M. L. Amicosante G. Frere J. M.. (1999). Structure of In31, a blaIMP-containing Pseudomonas aeruginosa integron phyletically related to In5, which carries an unusual array of gene cassettes. Antimicrob. Agents Chemother. 43, 890–901. doi: 10.1128/AAC.43.4.890, PMID: 10103196 PMC89222

[ref12] Lombardi G. Luzzaro F. Docquier J. D. Riccio M. L. Perilli M. Coli A.. (2002). Nosocomial infections caused by multidrug-resistant isolates of pseudomonas putida producing VIM-1 metallo-beta-lactamase. J. Clin. Microbiol. 40, 4051–4055. doi: 10.1128/JCM.40.11.4051-4055.2002, PMID: 12409373 PMC139695

[ref13] Marchetti V. M. Bitar I. Sarti M. Fogato E. Scaltriti E. Bracchi C.. (2021). Genomic characterization of VIM and MCR co-producers: the first two clinical cases, in Italy. Diagnostics (Basel) 11:0079. doi: 10.3390/diagnostics11010079, PMID: 33418979 PMC7825325

[ref14] Mazel D. (2006). Integrons: agents of bacterial evolution. Nat. Rev. Microbiol. 4, 608–620. doi: 10.1038/nrmicro1462, PMID: 16845431

[ref15] Mazel D. Davies J. (1999). Antibiotic resistance in microbes. Cell. Mol. Life Sci. 56, 742–754. doi: 10.1007/s000180050021 11212334 PMC11147152

[ref16] Miriagou V. Papagiannitsis C. C. Kotsakis S. D. Loli A. Tzelepi E. Legakis N. J.. (2010). Sequence of pNL194, a 79.3-kilobase IncN plasmid carrying the blaVIM-1 metallo-beta-lactamase gene in Klebsiella pneumoniae. Antimicrob. Agents Chemother. 54, 4497–4502. doi: 10.1128/AAC.00665-10, PMID: 20660690 PMC2944605

[ref17] Miriagou V. Tzelepi E. Gianneli D. Tzouvelekis L. S. (2003). Escherichia coli with a self-transferable, multiresistant plasmid coding for metallo-beta-lactamase VIM-1. Antimicrob. Agents Chemother. 47, 395–397. doi: 10.1128/AAC.47.1.395-397.2003, PMID: 12499222 PMC149029

[ref18] Moura A. Soares M. Pereira C. Leitao N. Henriques I. Correia A. (2009). INTEGRALL: a database and search engine for integrons, integrases and gene cassettes. Bioinformatics 25, 1096–1098. doi: 10.1093/bioinformatics/btp105, PMID: 19228805

[ref19] Naas T. Cuzon G. Villegas M. V. Lartigue M. F. Quinn J. P. Nordmann P. (2008). Genetic structures at the origin of acquisition of the beta-lactamase Bla KPC gene. Antimicrob. Agents Chemother. 52, 1257–1263. doi: 10.1128/AAC.01451-07, PMID: 18227185 PMC2292522

[ref20] Papagiannitsis C. C. Dolejska M. Izdebski R. Giakkoupi P. Skalova A. Chudejova K.. (2016). Characterisation of IncA/C2 plasmids carrying an In416-like integron with the blaVIM-19 gene from Klebsiella pneumoniae ST383 of Greek origin. Int. J. Antimicrob. Agents 47, 158–162. doi: 10.1016/j.ijantimicag.2015.12.001, PMID: 26795022

[ref21] Papagiannitsis C. C. Paskova V. Chudejova K. Medvecky M. Bitar I. Jakubu V.. (2018). Characterization of pEncl-30969cz, a novel ColE1-like plasmid encoding VIM-1 carbapenemase, from an Enterobacter cloacae sequence type 92 isolate. Diagn. Microbiol. Infect. Dis. 91, 191–193. doi: 10.1016/j.diagmicrobio.2018.01.024, PMID: 29477275

[ref22] Papagiannitsis C. C. Studentova V. Ruzicka F. Tejkalova R. Hrabak J. (2013). Molecular characterization of metallo-beta-lactamase-producing Pseudomonas aeruginosa in a Czech hospital (2009-2011). J. Med. Microbiol. 62, 945–947. doi: 10.1099/jmm.0.056119-0, PMID: 23493029

[ref23] Papousek I. Papagiannitsis C. C. Medvecky M. Hrabak J. Dolejska M. (2017). Complete nucleotide sequences of two VIM-1-encoding plasmids from Klebsiella pneumoniae and Leclercia adecarboxylata isolates of Czech origin. Antimicrob. Agents Chemother. 61:2648. doi: 10.1128/AAC.02648-16, PMID: 28264839 PMC5404563

[ref24] Partridge S. R. Hall R. M. (2003). The IS1111 family members IS4321 and IS5075 have subterminal inverted repeats and target the terminal inverted repeats of Tn21 family transposons. J. Bacteriol. 185, 6371–6384. doi: 10.1128/JB.185.21.6371-6384.2003, PMID: 14563872 PMC219399

[ref25] Poirel L. Heritier C. Tolun V. Nordmann P. (2004). Emergence of oxacillinase-mediated resistance to imipenem in Klebsiella pneumoniae. Antimicrob. Agents Chemother. 48, 15–22. doi: 10.1128/AAC.48.1.15-22.2004, PMID: 14693513 PMC310167

[ref26] Ren Y. Ren Y. Zhou Z. Guo X. Li Y. Feng L.. (2010). Complete genome sequence of Enterobacter cloacae subsp. cloacae type strain ATCC 13047. J. Bacteriol. 192, 2463–2464. doi: 10.1128/JB.00067-10, PMID: 20207761 PMC2863489

[ref27] Rotova V. Papagiannitsis C. C. Skalova A. Chudejova K. Hrabak J. (2017). Comparison of imipenem and meropenem antibiotics for the MALDI-TOF MS detection of carbapenemase activity. J. Microbiol. Methods 137, 30–33. doi: 10.1016/j.mimet.2017.04.003, PMID: 28390706

[ref28] Sheppard A. E. Stoesser N. Wilson D. J. Sebra R. Kasarskis A. Anson L. W.. (2016). Nested Russian doll-like genetic mobility drives rapid dissemination of the Carbapenem resistance gene blaKPC. Antimicrob. Agents Chemother. 60, 3767–3778. doi: 10.1128/AAC.00464-16, PMID: 27067320 PMC4879409

[ref29] Tato M. Coque T. M. Baquero F. Canton R. (2010). Dispersal of carbapenemase blaVIM-1 gene associated with different Tn402 variants, mercury transposons, and conjugative plasmids in Enterobacteriaceae and Pseudomonas aeruginosa. Antimicrob. Agents Chemother. 54, 320–327. doi: 10.1128/AAC.00783-09, PMID: 19901094 PMC2798558

[ref30] Yong D. Toleman M. A. Giske C. G. Cho H. S. Sundman K. Lee K.. (2009). Characterization of a new metallo-beta-lactamase gene, Bla(NDM-1), and a novel erythromycin esterase gene carried on a unique genetic structure in Klebsiella pneumoniae sequence type 14 from India. Antimicrob. Agents Chemother. 53, 5046–5054. doi: 10.1128/AAC.00774-09, PMID: 19770275 PMC2786356

