# Supplementary figures and images for: Implication of different replicons in the spread of the VIM-1-encoding integron, In110, in Enterobacterales from Czech hospitals
Source: Front Microbiol. 2023 Jan 4;13:993240. doi: 10.3389/fmicb.2022.993240 (PMC9845580; doi:10.3389/fmicb.2022.993240)

## Slide 1
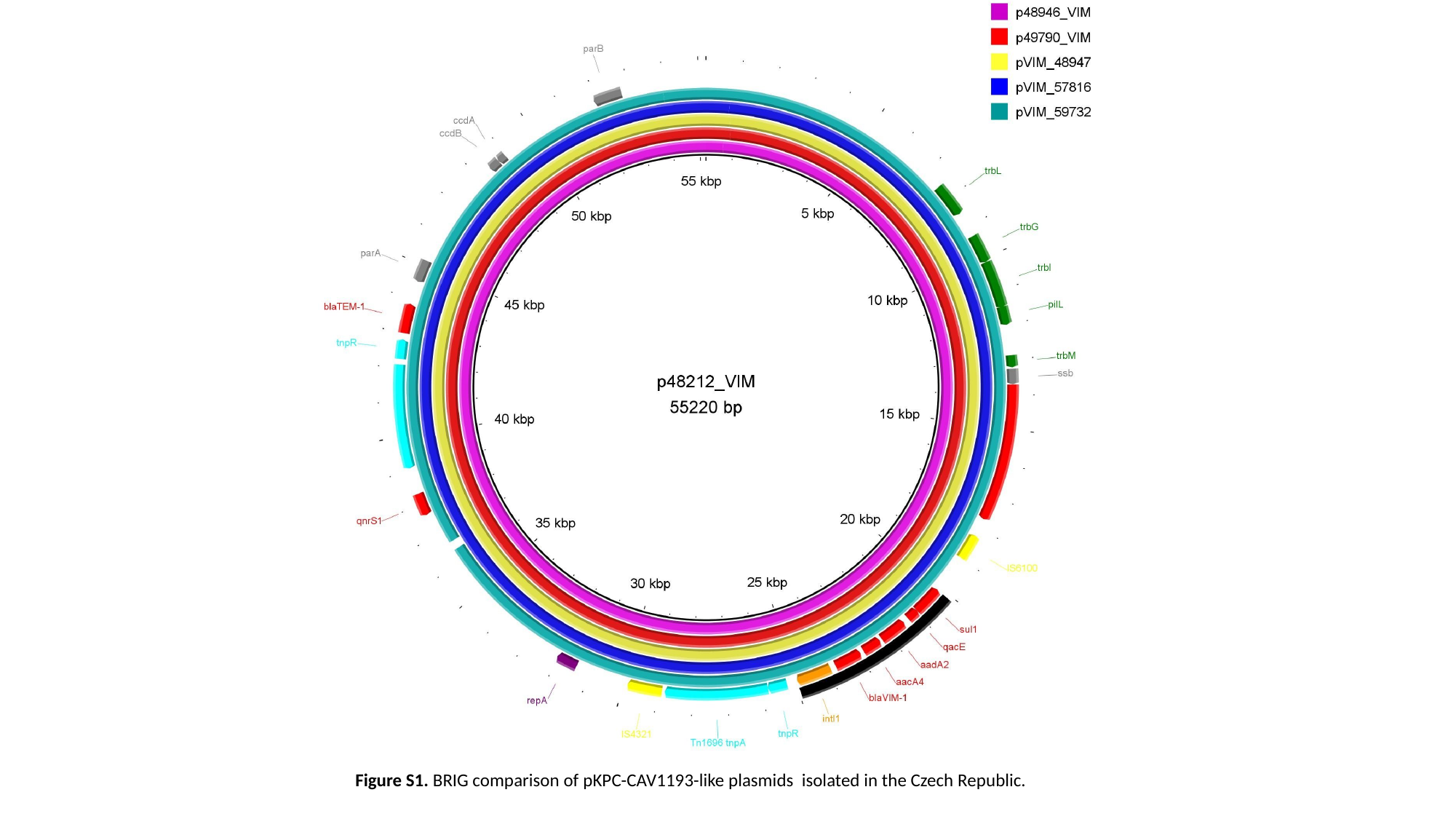

Figure S1. BRIG comparison of pKPC-CAV1193-like plasmids isolated in the Czech Republic.

Supplement: Supplementary file 1 [file Presentation_1.PPTX]

## Slide 1
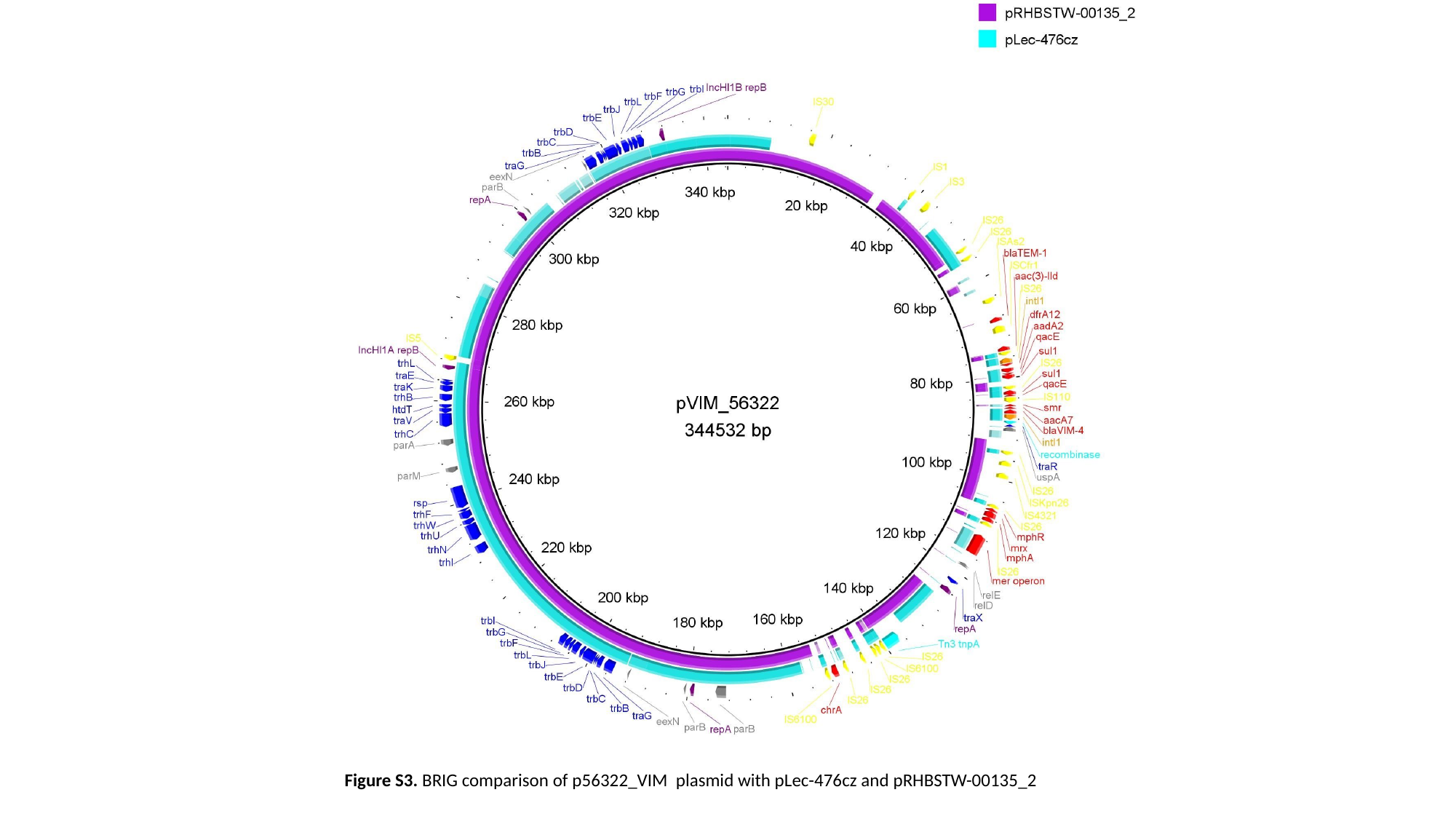

Figure S3. BRIG comparison of p56322_VIM plasmid with pLec-476cz and pRHBSTW-00135_2

Supplement: Supplementary file 3 [file Presentation_3.PPTX]
