# Supplementary material for: Implication of different replicons in the spread of the VIM-1-encoding integron, In110, in Enterobacterales from Czech hospitals
Source: Front Microbiol. 2023 Jan 4;13:993240. doi: 10.3389/fmicb.2022.993240 (PMC9845580; doi:10.3389/fmicb.2022.993240)
Supplement: Supplementary file 5 [file Presentation_5.PPTX]

## Slide 1
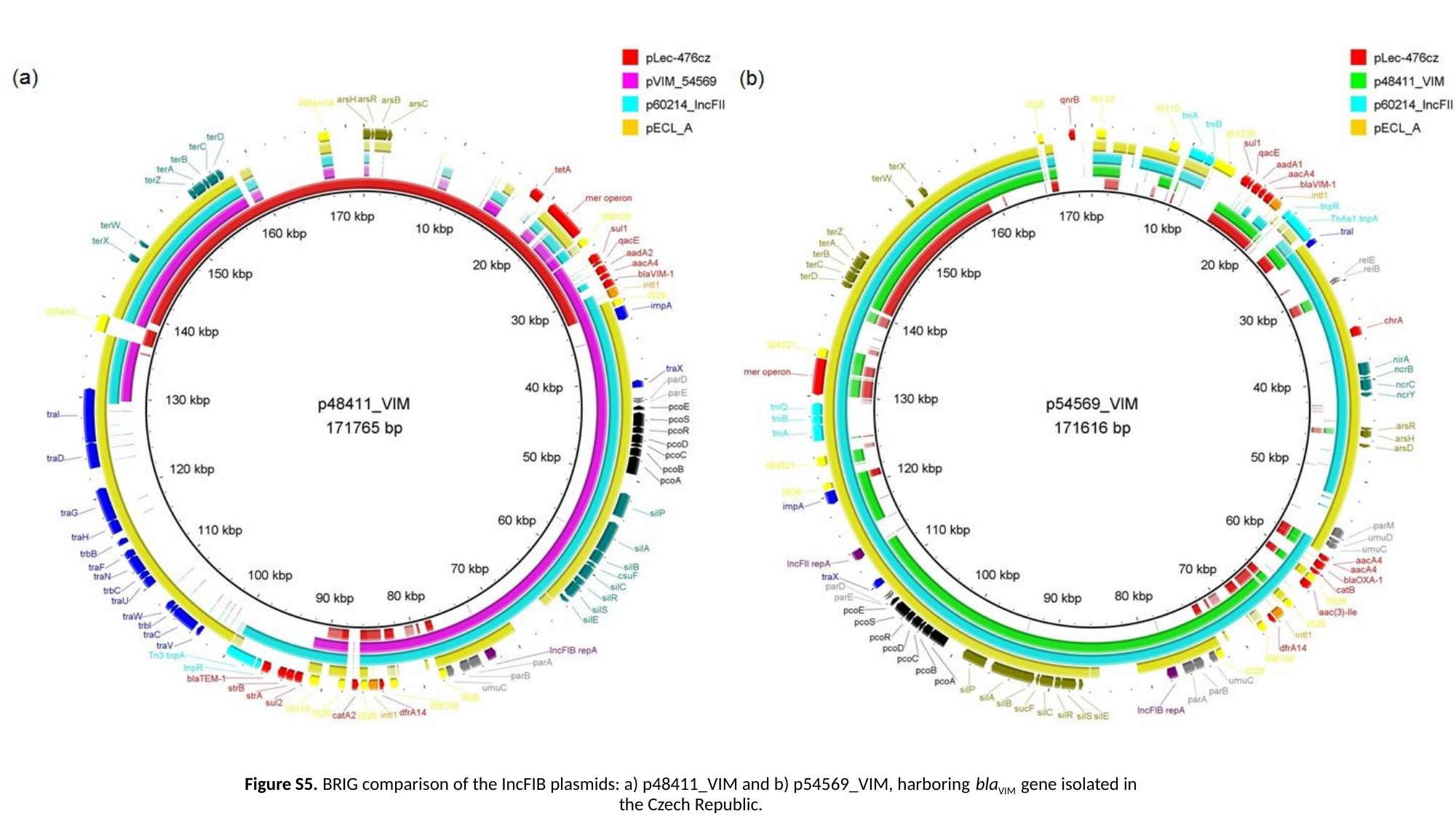

Figure S5. BRIG comparison of the IncFIB plasmids: a) p48411_VIM and b) p54569_VIM, harboring blaVIM gene isolated in the Czech Republic.
