# Supplementary material for: Implication of different replicons in the spread of the VIM-1-encoding integron, In110, in Enterobacterales from Czech hospitals
Source: Front Microbiol. 2023 Jan 4;13:993240. doi: 10.3389/fmicb.2022.993240 (PMC9845580; doi:10.3389/fmicb.2022.993240)
Supplement: Supplementary file 7 [file Presentation_7.PPTX]

## Slide 1
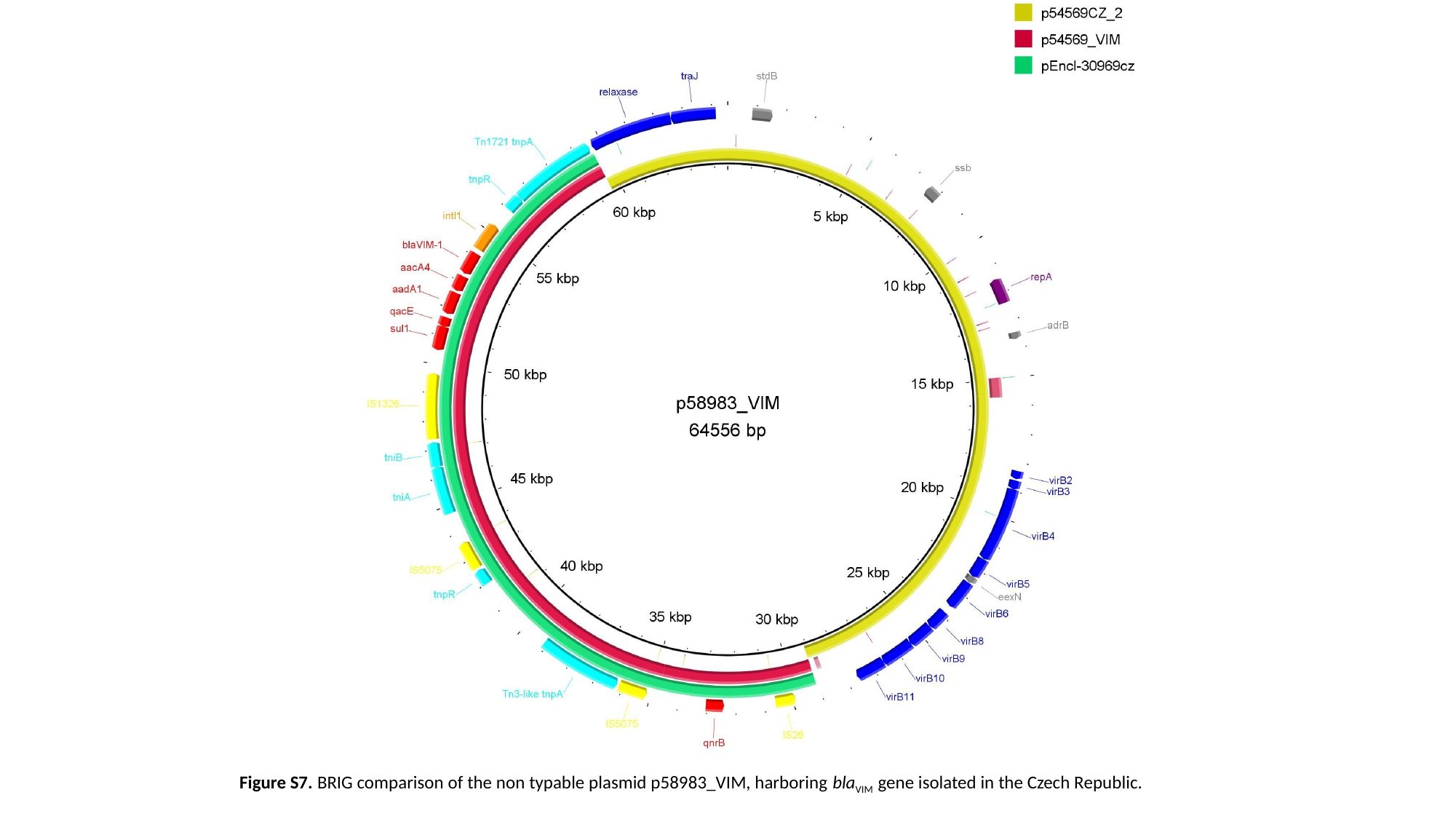

Figure S7. BRIG comparison of the non typable plasmid p58983_VIM, harboring blaVIM gene isolated in the Czech Republic.
